# The under-recognition and significance of frailty syndrome – a geriatric and forensic conundrum

**DOI:** 10.1007/s12024-024-00900-w

**Published:** 2024-10-05

**Authors:** Roger W. Byard

**Affiliations:** School of Biomedicine, The University of Adelaide, and Forensic Science SA Adelaide, Level 2, Room N237, Helen Mayo North, Frome Road, Adelaide, SA 5001 Australia

**Keywords:** Frailty syndrome, Autopsy, Cause of death, Weight loss, Sarcopenia, Cardiovascular disease, Osteoporosis

## Abstract

Frailty syndrome occurs in elderly individuals with declining muscle mass (sarcopenia), unintentional weight loss, decreasing physical strength and activity, exhaustion, and slow ambulation. It significantly increases morbidity and mortality with cardiovascular, renal disease and neurological disorders, osteoporosis and fractures, endocrine and immunological dysfunction and a variety of malignancies. It is increasing in incidence as the population ages. However, unfortunately as identification relies on clinical and not pathological evaluations, its contribution to a wide range of comorbidities and its role in terminal episodes may not be recognized in a forensic context.

## Introduction

Frailty syndrome is a diagnosis characterised by specific manifestations with an increased risk for a number of quite disparate comorbidities [1]. Unfortunately the diagnosis relies on clinical evaluation and so it is an entity that cannot be identified on pathological grounds [2]. This means that medicolegal death registers will in all likelihood underestimate the incidence in an aging population. This is unfortunate as the relationship between frailty syndrome and significant adverse health outcomes may be of considerable forensic significance [3].

## Characteristics

The syndrome is characterized by a decrease in muscle mass (sarcopenia), unintentional weight loss, reduced physical strength and activity, exhaustion, and slow ambulation. It affects multiple systems increasing morbidity and mortality in the elderly associated with osteoporosis and fractures, cardiovascular and renal disease, neurological conditions including dementia, immunological dysfunction, diabetes mellitus, and malignancy [2, 4, 5].

## Diagnosis

Clinical guidelines have been formulated to assist with the diagnosis with a screening tool known as FRAIL [6, 7] which assesses *F*atigue, *R*esistance (inability to walk up a flight of stairs), *A*mbulation (inability to walk a short distance), *I*llness (> five comorbid conditions), and *L*oss of body mass (> 5% of total bodyweight) [1]. The frailty index measures the extent of deficits and gives some guidance to prognosis. For example, after controlling for educational level, sex and age, a one degree change annually in the frailty index has an 85% increased risk of disability and a five times increased risk of death [6]. The increased susceptibility to stressors has been likened to a domino effect that increases mortality and may involve conditions that would not normally be considered particularly significant in the healthy [8]. Sometimes “pre-fail” is used for individuals with only one or two of the above features. Unfortunately other definitions have been suggested [9–11], all of which contribute to difficulties with recognition at the time of autopsy.

## Incidence

The advancing age of many populations globally means that frailty will increasingly underpin many lethal conditions seen in forensic facilities. For example, in Australia the numbers of individuals aged *≥* 65 years has increased from 1 million (8.3% of the population) in 1970, to 2.1 million (12%) in 1995 and 4.2 million (16%). in 2020. It is predicted that this will increase to 21 to 23% by 2066 [12]. Similarly in Japan the 7.38 million individuals > 65 years of age is predicted to rise to 11 million by 2050 [13]. Globally the precentage of those aged 65 years and over is expected to rise from 10% in 2022 to 16% in 2050 [14], and in 2020 the countries with the highest percentages of this age group were Japan (28%), Italy (23%) and Portugal and Greece (21.8%) [15].

The incidence of frailty syndrome even in clinical practice may be difficult to accurately determine as there may not be effective monitoring and recording of cases. In the United States approximately 6–25% of individuals aged over 65 years have frailty syndrome with higher rates in minority groups and females [6, 16–18]. Frail individuals are three to five times more likely to die than the non- or prefrail [19]. The etiology remains unclear, however low grade inflammation with increased cytokine production and elevated levels of proinflammatory factors, hormonal changes, and activation of coagulation pathways with cellular senescence have all been demonstrated [1, 20–22].

## Issues at autopsy

Indications of frailty at autopsy include loss of muscle mass with a low body mass index (BMI). Sarcopenia, which is a lean body mass that is two standard deviations or more below the mean, manifests as reduced muscle bulk with fatty infiltration. Both of these factors compromise strength resulting in reduced mobility and general deconditioning with an increased risk for developing pressure ulcers. Significant weight loss may also involve anorexia and insulin resistance with abnormal levels of appetite control peptides such as leptin and neuropeptide Y. Obesity, which is also on the increase can, however, mask the usual phenotype [23]. As there has been a link reported between elder abuse and frailty syndrome the possibility of neglect or inflicted injury should always be considered. Pressure ulcers may be a manifestation of inadequate care [24].

Frailty syndrome should also be suspected if there are comorbidities such as hypertension and osteoarthritis [6] with their particular cardiovascular and orthopedic consequences. Other associated conditions include insulin resistance and diabetes mellitus, chronic renal failure possibly due to underlying thyroid hormone abnormalities, and marked coronary artery disease and cardiac failure [4, 25]. Frail individuals are often anemic due to iron deficiency and chronic disease [6]. Significant osteoporosis is often present with the risk of hip fracture being 25 times greater in elderly women with frailty and a history of falls [6, 26–28]. Frailty has also been linked to adverse post-operative outcomes with an increased incidence of sepsis [29, 30] and hypothermia may be an issue in the elderly with low BMIs and social isolation [31]. Unfortunately these associated conditions may not only cause frailty, but they may be caused by frailty, thus contributing to a downward spiral.

Formal neuropathological evaluation should be considered in the frail as there are higher rates of Parkinson disease [6] with four times the rate of Alzheimer disease and six times the rate of vascular dementia [32]. A history of depression is often found in those with frailty syndrome, related to social isolation and adverse economic factors and has been associated with an increase in the incidence of suicide [33].

The high incidence of underlying medical conditions in the frail may result in consumption of a multitude of prescription, non-prescription and herbal medications [6] with the significant potential for adverse drug interactions and reactions in addition to falls [2] Although toxicological screening in the ill elderly presents challenges in interpretation, it may nontheless be useful [34].

## Conclusion

Frailty syndrome is a complex entity marked by malnutrition and sarcopenia. However, although a low BMI may be strongly suggestive of the syndrome at autopsy [35, 36], the only way to determine whether the condition was a contributing factor to death is by carefully evaluating the medical history of an elderly decedent looking for formal assessments of the level of activity, physical strength and ambulation. Failure of clinicians to make the diagnosis, and/or lack of access to medical records, will unfortunately ensure that this remains an under-recognised entity in forensic circles as there are no diagnostic pathological features. This, in turn, will result in the full mechanisms underlying certain geriatric deaths not being completely and accurately delineated or understood.

### Key points

1. Frailty syndrome is most often found in elderly individuals.
2. Characteristics include declining muscle mass (sarcopenia), unintentional weight loss, decreased physical strength and activity, exhaustion, and slow ambulation.
3. It has significant associations with cardiovascular, renal disease and neurological disorders, osteoporosis and fractures, endocrine and immunological dysfunction, and a variety of malignancies.
4. Unfortunately its contribution to a wide range of comorbidities and to terminal episodes may not be recognized at autopsy as the diagnosis relies on clinical, not pathological parameters.
